# Supplementary material for: Association of G-Protein-Coupled Receptors autoantibodies with vasoregulation in Post-COVID
Source: PLoS One. 2026 Feb 24;21(2):e0343264. doi: 10.1371/journal.pone.0343264 (PMC12931808; doi:10.1371/journal.pone.0343264)
Supplement: Attachment — Submitted filename: Response to Reviewers.docx [file pone.0343264.s002.docx]

**RESPONSE TO REVIEWERS**

Dear Editor,

Thank you very much for handling our manuscript and for the reviewers’ insightful comments. We have carefully addressed all points raised, as detailed below.

**Editor Comments**

*When submitting your revision, we need you to address these additional requirements.*

*1.Please ensure that your manuscript meets PLOS ONE's style requirements, including those for file naming. The PLOS ONE style templates can be found at* [*https://journals.plos.org/plosone/s/file?id=wjVg/PLOSOne_formatting_sample_main_body.pdf*](https://journals.plos.org/plosone/s/file?id=wjVg/PLOSOne_formatting_sample_main_body.pdf) *and* [*https://journals.plos.org/plosone/s/file?id=ba62/PLOSOne_formatting_sample_title_authors_affiliations.pdf*](https://journals.plos.org/plosone/s/file?id=ba62/PLOSOne_formatting_sample_title_authors_affiliations.pdf)

**ANSWER:** We have carefully revised our submission to ensure full compliance with PLOS ONE’s style requirements. The manuscript has been reformatted according to the provided templates, and all files have been renamed following the journal’s conventions.

*In this instance it seems there may be acceptable restrictions in place that prevent the public sharing of your minimal data. However, in line with our goal of ensuring long-term data availability to all interested researchers, PLOS’ Data Policy states that authors cannot be the sole named individuals responsible for ensuring data access (*[*http://journals.plos.org/plosone/s/data-availability#loc-acceptable-data-sharing-methods*](http://journals.plos.org/plosone/s/data-availability#loc-acceptable-data-sharing-methods)*).Data requests to a non-author institutional point of contact, such as a data access or ethics committee, helps guarantee long term stability and availability of data. Providing interested researchers with a durable point of contact ensures data will be accessible even if an author changes email addresses, institutions, or becomes unavailable to answer requests.Before we proceed with your manuscript, please also provide non-author contact information (phone/email/hyperlink) for a data access committee, ethics committee, or other institutional body to which data requests may be sent. If no institutional body is available to respond to requests for your minimal data, please consider if there any institutional representatives who did not collaborate in the study, and are not listed as authors on the manuscript, who would be able to hold the data and respond to external requests for data access? If so, please provide their contact information (i.e., email address). Please also provide details on how you will ensure persistent or long-term data storage and availability.*

**ANSWER:** Thank you for Your message regarding the data availability requirements. We would like to confirm that all relevant data underlying the findings of our manuscript have been deposited in the Figshare repository. The dataset is publicly available and can be accessed at the following DOI: https://doi.org/10.6084/m9.figshare.30127723.

This ensures persistent accessibility of the data, independent of the authors. Our Data Availability Statement has been updated accordingly.

**Reviewer #1**

*This study is a follow up of the same group showing a correlation between vasoregulatory GPCR autoantibodies and neuronal disordered. Authors should compare autoantibodies in these two studies, the published one and the present one and see if autoantibodies to GPCR correlates similarly with neuronal symptoms and blood pressure variations.*

**ANSWER:** We thank the reviewer for this thoughtful comment. The present manuscript, however, investigates a newly recruited Post-COVID population that is distinct from the cohort described in our previous publication. Since the participants are not overlapping and clinical assessments were performed independently, a direct follow-up or combined analysis is unfortunately not feasible. Nevertheless, we acknowledge the reviewer’s suggestion and agree that future longitudinal studies including both patient populations would be valuable to further clarify the relationship between GPCR autoantibodies, neuronal symptoms, and blood pressure regulation

*Patients need to be better described: interval between acute COVID and sampling; symptoms developed by Long COVID patients and their absence in Controls, comorbidities and as well as history of autoimmunity in the two groups, etc… and give frequencies in the cohort. Then they could ask if autoantibody (or one type of autoantobody) concentration correlates with some specific symptoms?*

**ANSWER:** We thank the reviewer for this important comment. We have included the description of the main neurological symptoms using the CERAD-Plus score, which provides a standardized assessment of neurocognitive performance. Comorbidities were already reported in the manuscript, and history of autoimmunity (GPCR autoantibodies) has been devoted a separate table (Table 2) for clarity. With regard to correlation analyses, these were indeed the main focus of our previous publication. In the present study, we intended to focus specifically on vascular symptoms, therefore, correlations with neurological symptoms were not included here.

Although correlation shown in Figure 1 are statistically valid, the intensity of the correlation is not high. One question then the relevance of the correlation. Does this figure include only Long COVID patients? Or also Covid resolved one?

**ANSWER:** We thank the reviewer for this observation. Figure 1 includes only Post COVID patients. We acknowledge that the correlation strength is moderate. Nevertheless, it is statistically significant and highlights a potential link between GPCR autoantibody levels and vascular measures in the Post-COVID population. In order to enhance transparency, we added the absolute values of FMD in a revised version of Table 2. We also corrected for covariates and attenuates the statement about the potential association for EDNRA with FMD. We have also added a clarifying statement in the figure legend and the Results section to explicitly note the patient group included.

Figure 1 and 2: Authors should present the same correlation of COVID recovered patients, especially as the 22% of Covid recovered with autoantibodies have concentration similar to COVID recovered ones, apart for ADRB2 which concentration is much higher in COVID recovered vs long COVID. Authors should comment also this latest difference.

**ANSWER:** We thank the Reviewer for this important remark. In response, we have added an additional graph to Figure 1, illustrating the non-significant correlation in the non Post-COVID group. As also outlined in the Discussion, one limitation of our study is the lack of sufficient central blood-pressure data in the control group. Central aortic blood-pressure measurements were only available in 9 of 54 non-Post-COVID subjects, which makes these data insufficiently powered and therefore not suitable for display alongside the main results. For completeness, however, we report the available baseline central blood-pressure values for You below:

|  | Post-COVID (IQR) | No Post-COVID (IQR) | *p* |
| --- | --- | --- | --- |
| Central systolic blood pressure (mmHg) | 126 (113-136) | 125 (109-143) | 0.7087 |
| Central diastolic blood pressure (mmHg) | 81 (74-90) | 76 (71-85) | 0.2822 |

We discussed the data about ADRB2 excess in GPCR positive patients in the non Post-COVID population in the revised version of the manuscript.

**REVIEWER #2**

A) Regarding the abstract:
In the results section, the authors state that “There was no significant association of any GPCR autoantibody concentration with FMD or Lp-PLA2 in the control group,” but later in the manuscript they mention that no association was found in either group, not only in the controls. This discrepancy should be clarified.

**ANSWER:** We rechecked the mentioned passage from the abstract, but we were not able to identify where a contradictory statement can be found in the subsequent text. Perhaps you were referring to the following section in the Discussion: ‘*Central aortic blood pressure and FMD are markers of arterial function. The present study also investigated capillary function by means of nailfold microscopy in post-COVID patients. The findings did not reveal an association between GPCR autoantibody concentrations and capillary function.’* However, capillary function is distinct from arterial function and therefore cannot be considered an expression of FMD.

B) Regarding the results:
1. The authors report a positive association between EDNRA autoantibodies and FMD. However, is FMD significantly different between the Post-COVID and the non-Post-COVID groups? A comparison of FMD values between these two groups should be presented first. Only then should the association between EDNRA and FMD in the Post-COVID group be introduced. If no difference exists between the Post-COVID and control group FMD,
a) Is the observed association between FMD and autoantibodies clinically relevant?
b) Could this association be merely reflecting a third, unaccounted-for confounding variable (If no significant differences in FMD are observed between the groups)?

**ANSWER:** We thank the Reviewer for this valuable comment. Although no statistically significant difference was observed between the groups, there was a numerical trend toward lower FMD, indicating attenuated arterial function in the Post-COVID group. To further investigate this potential association, we performed a Spearman correlation analysis, as presented in the original version of the manuscript. The Results and Table 2 have been updated with FMD data to specifically address the Reviewer’s remark. However, regression analysis did not retain statistical significance after adjustment for covariates (p=0.0745), that have been identified as significantly different between both groups, namely BMI, vaccination status, and sex. The results of the multivariate analysis are mentioned in the Results, and the statement about the correlation analysis has been attenuated in the revised version of the manuscript.

2. Similarly to 1., the authors report a negative association between AGTR2, ADRB1, and ADRB2 autoantibody levels and central systolic blood pressure, as well as a negative association between ADRB1 and CHMR4 autoantibodies and central diastolic blood pressure. However, do baseline vascular parameters differ between the Post-COVID and non-Post-COVID groups? If no data from the control group are available, how do the vascular parameters of the Post-COVID group compare to “normal/expected” values reported in the literature?

**ANSWER:** We thank the Reviewer for this important question. Unfortunately, the available central aortic blood-pressure data for the non-Post-COVID group are limited (9 of 54 subjects), so these control data are underpowered for robust between-group inference and have therefore not been displayed. For completeness, we report the baseline central blood-pressure values below:

|  | Post-COVID (IQR) | No Post-COVID (IQR) | *p* |
| --- | --- | --- | --- |
| Central systolic blood pressure (mmHg) | 126 (113-136) | 125 (109-143) | 0.7087 |
| Central diastolic blood pressure (mmHg) | 81 (74-90) | 76 (71-85) | 0.2822 |

As noted in the Discussion, the limited central blood pressure data of the non-Post-COVID subgroup constitutes a limitation of our study. Central systolic blood pressure is generally defined as hypertensive above 130 mmHg (Cheng HM et al. *J Am Coll Cardiol* 2013;62:1780‐1787). The median central systolic pressure in our cohort, the data displayed to you, did not exceed this threshold.

3. The authors state that no association was found between Lp-PLA2 levels and Post-COVID symptoms. In the Materials and Methods section, it is mentioned that Lp-PLA2 has been correlated with COVID-19 severity. Was any subgroup analysis performed based on the severity of the acute COVID-19 infection in the Post-COVID group? For example, was an association explored between Lp-PLA2 levels and individuals who had mild COVID-19, and separately for those with moderate COVID-19? The lack of subgroup analysis for potential confounding variables (e.g., COVID-19 severity, vaccination status) could represent a limitation of the study. Such an analysis should be conducted, or otherwise acknowledged as a limitation of the study.

**ANSWER**: We thank the Reviewer for this insightful comment. We agree that disease severity and vaccination status represent potential confounding variables that could influence Lp-PLA2 levels. This has been added to the limitation section of the Discussion.

4. The authors mention that no association was found between GPCR autoantibodies and functional or morphological microcirculatory changes. It would be valuable to include figures or tables that represent these negative findings.

**ANSWER:** Morphological microcirculatory changes have been provided in the Supplementary Material.

C) Regarding the discussion:
The limitations section should consider the possibility that other variables may account for the observed associations.

**ANSWER:** We have revised the Discussion accordingly to acknowledge that other unmeasured variables may account for the observed associations.

D) 1.- In Table 3, the table legend seems inconsistent with the table content. It states “numbers in brackets indicate…”, but the table does not contain any brackets.

**ANSWER:** We thank the Reviewer for this remark. The legend has been corrected accordingly in the revised manuscript.

E) Regarding grammar and typography: There are minor typographical and grammatical errors throughout the manuscript. For example: in the abstract, the word “BACKGROUD” should be corrected to “BACKGROUND”. In Table 1, capitalization should be standardized for terms such as sex, chronic obstructive pulmonary disease, hypercholesterinemia, and smoking. The word “Comborbidities” should be corrected to “Comorbidities”.

**ANSWER:** We thank the Reviewer for the careful reading. All typographical and formatting issues mentioned have been corrected in the revised version of the manuscript.
